# The effect of human settlement on the abundance and community structure of ammonia oxidizers in tropical stream sediments

**DOI:** 10.3389/fmicb.2015.00898

**Published:** 2015-08-31

**Authors:** Mariana P. Reis, Marcelo P. Ávila, Rosalinde M. Keijzer, Francisco A. R. Barbosa, Edmar Chartone-Souza, Andréa M. A. Nascimento, Hendrikus J. Laanbroek

**Affiliations:** ^1^Departamento de Biologia Geral, Instituto de Ciências Biológicas, Universidade Federal de Minas GeraisBelo Horizonte, Brazil; ^2^Department of Microbial Ecology, Netherlands Institute of EcologyWageningen, Netherlands; ^3^Institute of Environmental Biology, Utrecht UniversityUtrecht, Netherlands

**Keywords:** ammonia oxidizers, *amoA* gene, 16S rRNA, qPCR, DGGE fingerprint, freshwater sediment, human settlement activities, niche speciation

## Abstract

Ammonia-oxidizing archaea (AOA) and ammonia-oxidizing bacteria (AOB) are a diverse and functionally important group in the nitrogen cycle. Nevertheless, AOA and AOB communities driving this process remain uncharacterized in tropical freshwater sediment. Here, the effect of human settlement on the AOA and AOB diversity and abundance have been assessed by phylogenetic and quantitative PCR analyses, using archaeal and bacterial *amoA* and 16S rRNA genes. Overall, each environment contained specific clades of *amoA* and 16S rRNA genes sequences, suggesting that selective pressures lead to AOA and AOB inhabiting distinct ecological niches. Human settlement activities, as derived from increased metal and mineral nitrogen contents, appear to cause a response among the AOB community, with *Nitrosomonas* taking advantage over *Nitrosospira* in impacted environments. We also observed a dominance of AOB over AOA in mining-impacted sediments, suggesting that AOB might be the primary drivers of ammonia oxidation in these sediments. In addition, ammonia concentrations demonstrated to be the driver for the abundance of AOA, with an inversely proportional correlation between them. Our findings also revealed the presence of novel ecotypes of Thaumarchaeota, such as those related to the obligate acidophilic *Nitrosotalea devanaterra* at ammonia-rich places of circumneutral pH. These data add significant new information regarding AOA and AOB from tropical freshwater sediments, albeit future studies would be required to provide additional insights into the niche differentiation among these microorganisms.

## Introduction

Nitrification, in which ammonia is converted to nitrite and subsequently to nitrate, is a key process in the global nitrogen cycle that is essential for the functioning of many ecosystems. Aerobic ammonia oxidation, the first step in nitrification, converts reduced inorganic nitrogen species to oxidized ones (Gruber and Galloway, 2008). This process is mediated by autotrophic ammonia-oxidizing bacteria (AOB) (Purkhold et al., 2000), and also by autotrophic ammonia-oxidizing archaea (AOA) of the Thaumarchaeota phylum (Wuchter et al., 2006).

The environmental drivers that shape the structure of microbial communities provide fundamental insight in the maintenance of biodiversity and in the function that they underpin. The distribution and/or abundance of AOA and AOB is thought to be influenced mainly by pH (Nicol et al., 2004; He et al., 2007; Daebeler et al., 2012), temperature (Tourna et al., 2008), ammonium availability (Martens-Habbena et al., 2009; Lehtovirta-Morley et al., 2011; Tourna et al., 2011), oxygen accessibility (Coolen et al., 2007; Lam et al., 2009; Daebeler et al., 2012), and metal concentrations (Stephen et al., 1999; Li et al., 2009; Mertens et al., 2009; Vasileiadis et al., 2012; Liu et al., 2014). Interestingly, many studies have revealed the dominance of AOA over AOB in many environments (Francis et al., 2005; Leininger et al., 2006; Wuchter et al., 2006; Zhang et al., 2008; Li et al., 2009). However, AOB seem to be more abundant in metal-polluted environments than AOA (Ruyters et al., 2013; Liu et al., 2014).

Although efforts have been made to understand the AOB and AOA community structures and their ecological roles, the effect of human settlement associated with tropical metal-mining on the diversity, abundance and distribution of these communities is still unknown. Herein, we hypothesize that such human settlement, which is also connected with mineral nitrogen pollution, would lead to different responses on the abundance and composition of AOA and AOB communities. From such different responses it might be able to detect some genera or groups as potential bioindicators of metal-mining pollution or of water quality. To address this hypothesis we used archaeal and bacterial *amoA* genes as a molecular marker together with Betaproteobacteria- and Thaumarchaeota-specific 16S rRNA gene-targeting primers, for tracking the distribution of AOA and AOB in tropical sediment streams with different metal concentrations due to historical pollution from metal and smelter activities. Moreover, we used the quantitative PCR approach to unveil the abundance of *amoA* genes among the prokaryotic community present in these freshwater sediment samples.

## Materials and Methods

### Study Area and Physicochemical Analysis

The Iron Quadrangle region (Minas Gerais, Brazil) is extremely rich in ores and has been historically explored. A total of six sites were sampled: three sites located in mining-impacted streams sediments, i.e., in the Mina stream (MS, 19°58′46.80″S and 43°49′17.07″W), in the Tulipa stream (TS, 19°59′08.1″S and 43°28′15.2″), and in the Carrapatos stream (CS, 19°58′15.4″S and 43°27′50.7″W); and the remaining sites from non-mining-impacted streams, i.e., two sites separated by 50 m distance from each other in a nameless stream (S1 and S2, 19°59′12.1″S and 43°29′27.5″W), and one site in the Mutuca stream (MTS, 20°00′37.23″S and 43°58′08.92″W). Collection of sediment samples has been previously described by our group (Reis et al., 2014), with exception of the MTS, which is not subjected to the influence of human settlement and belongs to the environmental protection area of the Minas Gerais state spring sanitation company. Only the names of the Mina stream and the Mutuca stream are formal names.

All the stream water samples were analyzed for temperature, pH, and dissolved oxygen (DO). Total nitrogen (TN), total phosphorus (TP), nitrite (NO_2_^-^-N), nitrate (NO_3_^-^-N), and soluble reactive phosphorus (PO_4_^3-^-P) were measured as previously described by Mackereth et al. (1978). The concentration of ammonia (NH_4_^+^-N) was measured according to Koroleff (1976). The results were presented in an earlier study (Reis et al., 2014), except for the MTS, which was physicochemically characterized in the present study (**Tables 1** and **2**). Total phosphorus concentrations allowed categorization of the streams as eutrophic (MS), mesotrophic (CS), oligotrophic (non-mining-impacted streams S1, S2, and MTS), and ultraoligotrophic (TS).

**Table 1:** Physicochemical characteristics of the bulk water samples.

	CW	TW	MW	SW1	SW2	MTW
**General characteristics**						
pH	7.91	6.53	6.20	6.66	6.50	6.00
Conductivity (μS cm^-1^)	227	1,500	2,151	13	13	1,852
Temperature (°C)	17	23	18	18	20	18
DO (mg O_2_ l^-1^)	12.5	3.6	9.1	12.1	12.1	9.1
**Chemical characteristics**						
Total P (μg l^-1^)	63.7	1.5	77.6	10.6	10.6	13.1
PO_4_-P (μg P l^-1^)	2.3	0.2	2.3	2.3	2.3	8.5
NH_4_^+^-N (μg N l^-1^)	122.7	1,600.0	829.5	25.1	25.1	12.5
NO_2_-N (μg N l^-1^)	0.5	12.5	161.3	0.2	0.2	0.2
NO_3_-N (μg N l^-1^)	6.0	13.3	3103.8	4.6	4.6	55.6
Reference	Reis et al. (2014)	Reis et al. (2014)	Reis et al. (2014)	Reis et al. (2014)	Reis et al. (2014)	In the present study

*CW, water from Carrapatos stream (CS); TW, water from Tulipa stream (TS); MW, water from Mina stream (MS); SW1 and SW2, water from sites 1 and 2 of the non-impacted mining stream; MTW, water from Mutuca stream (MTS).*

**Table 2:** Metal and metalloids concentrations of water and sediment samples (mg l^-1^ in water, mg kg^-1^ in sediments).

Metal and metalloids	CW	CS	TW	TS	SW1	SS1	SW2	SS2	MW	MS	MTW	MTS
As	0.14	<1.25	<0.05	<1.25	<0.05	<1.25	<0.05	<1.25	<0.10	297.10	0.01	0.15
Cr	<0.10	12.75	<0.10	<2.50	<0.10	<2.50	<0.10	6.25	<0.10	17.30	<0.01	<0.01
Cu	<0.05	5.75	<0.05	8.75	<0.05	9.50	<0.05	18.75	0.19	387.70	0.01	1.73
Fe	2.13	21,850.00	<0.05	437.50	0.11	2,136.00	0.11	5,238.00	0.52	492.80	<0.003	502.42
Mg	14.84	1,416.00	285.90	462.50	0.80	378.00	0.80	314.00	NT	NT	0.85	31.22
Mn	1.04	2,319.00	0.59	197.75	<0.05	1,41	<0.05	392.50	1.45	1,285.00	<0.002	82.56
Ni	<0.05	13.25	<0.05	2.50	<0.05	7.50	<0.05	6.25	<0.10	9.00	<0.01	<0.05
Pb	<0.10	3.50	<0.10	9.50	<0.10	2.75	<0.10	9.00	NT	8.70	<0.05	2.60
Zn	<0.05	24.50	<0.05	2.25	<0.05	5.75	<0.05	14.75	0.20	180.90	<0.003	15.92
Reference	Reis et al. (2014)		Reis et al. (2014)		Reis et al. (2014)		Reis et al. (2014)		Reis et al. (2014)		In the present study	

*NT, not tested; CW and CS, water and sediment from CS; TW and TS, water and sediment from TS; MW and MS, water and sediment from Mina stream; SW1, SW2, SS1, and SS2 water and sediment from sites 1 and 2 of the non-impacted mining stream, respectively; MTW and MTS, water and sediment from MTS.*

### DNA Extraction and Quantitative PCR (qPCR) Assay

Total DNA was extracted from the sediment samples (10 g wet weight) using the PowerMax soil DNA isolation kit (MoBio Laboratories, USA) according to the manufacturer’s instructions. The DNA samples were stored at –20°C until further processed. **Table 3** gives details of all the primers and the PCR conditions used in this study. The copy numbers of archaeal and bacterial *amoA* genes were quantified using the primer set Arch-amoAF and Arch-amoAR (Francis et al., 2005), and *amoA*-1F and *amoA*-2R (Rotthauwe et al., 1997; Stephen et al., 1999), respectively. Each reaction was performed in a 20 μL volume containing 10 ng of DNA, 1 μL of 5 μM of each primer and 10 μL of SYBR Green using the Rotor-Gene SYBR Green PCR Kit (Qiagen, Hilden, Germany), specific for the Rotor-Gene Q2 plex HRM Platform (Qiagen, Hilden, Germany). In the qPCR assay clones of *Escherichia coli* JM109 containing the archaeal and bacterial *amoA* gene fragments were included as standard, generating standard curves from seven dilutions. A control reaction without template DNA was included in each qPCR assay. All DNA samples and the negative control were analyzed in triplicate to obtain an accurate value for the *amoA* gene abundance in each sediment. Aliquots of the qPCR products were run on an agarose gel to identify unspecific PCR products such as primer dimers or fragments with unexpected lengths (data not shown). The amplifications efficiencies of AOB and AOA *amoA* gene were 90 and 109%, with *r*^2^-values of 0.999 and 0.992, respectively.

**Table 3:** Primer sequences and PCR conditions used.

Primer set	Temperature cycling	Primer sequences	Reference	Approach
21F/958R	20 cycles of 95°C for 30 s; 53–63°C (–0.5°C) for 45 s; 72°C for 60 s	21: 5′TTCCGGTTGATCCYGCCG GA3′ 958: 5′YCCGGCGTTGAMTCCAATT3′	DeLong (1992)	DGGE-PCR
Parch519/Arch915	35 cycles of 95°C for 30 s; 57°C for 30 s; 72°C for 45 s	Parch519: 5′CAGCCGCCGCGGTAA3′ **Arch915**: 5′GTGCTCCCCCGCCAATTCCT3′	Coolen et al. (2004)	DGGE-PCR
βAMOf /βAMOr	20 cycles of 95°C for 30 s; 55–65°C (–0.5°C) for 30 s; 72°C for 45 s	βAMOf: 5′TGGGGRATAACGCAYCGAAAG3′ βAMOr: 5′AGACTCCGATCCGGACTACG 3′	McCaig et al. (1994)	DGGE-PCR
CTO189f /CTO654r	35 cycles of 95°C for 30 s; 57°C for 30 s; 72°C for 45 s	**CTOf**: 5′GC-GGAGGAAAGCAGGGGATCG3′ CTOr: 5′CTAGCCTTTGTAGTTTCAAACGC3′	Kowalchuk et al. (1997)	DGGE-PCR
Arch-*amoA*F/Arch-amoAR	35 cycles of 95°C for 30 s; 56°C for 25 s; 72°C for 45 s	**Arch-*amoA*F**: 5′STAATGGTCTGGCTTAGACG3′ Arch-amoAR: 5′GCGGCCATCCATCTGTATGT3′	Francis et al. (2005)	DGGE-PCR and qPCR
AmoA-1F/*amoA*-2R	35 cycles of 95°C for 30 s; 55°C for 25 s; 72°C for 45 s	**AmoA-1F**: 5′GGGGTTTCTACTGGTGGT3′ *amoA*-2R: 5′CCCCTCKGSAAAGCCTTCTTC3′	Rotthauwe et al. (1997), Nicolaisen and Ramsing (2002)	DGGE-PCR
*amoA*-1F^∗^/*amoA*-2R	35 cycles of 95°C for 30 s; 55°C for 25 s; 72°C for 45 s	*amoA*-1F^∗^: 5′GGGGHTTYTACTGGTGGT3′ *amoA*-2R: sequence above	Stephen et al. (1999)	qPCR

*A 40-bp-long GC-clamp (5′CGCCCGCCGCGCCCCGCGCCCGGCCCGCCGCCCCCGCCCC3′; Muyzer et al., 1993) was attached to the 5′ end of the primers in bold to prevent complete melting of the PCR products during DGGE.*

*^∗^Forward primer designed by Rotthauwe et al. (1997), but with minor modification made by Stephen et al. (1999).*

### PCR-Denaturing Gradient Gel Electrophoresis (DGGE) Analysis

In attempt to assess the diversity and distribution of ammonia oxidizers, denaturing gradient gel electrophoresis (DGGE) of the AOA 16S rRNA gene was performed by a nested PCR with the archaeal primer set 21F and 958R followed by the thaumarchaeotal primer set Parch519F and Arch915R-GC, as previously described by Vissers et al. (2009). To detect the presence of archaeal *amoA* genes in the community the primer set Arch-*amoA*F and Arch-*amoA*R was used as described by Wuchter et al. (2006) and Vissers et al. (2012), (**Table 3**).

For the PCR-DGGE of the AOB 16S rRNA gene, a nested PCR was performed using the bacterial primer set βAMOf and βAMOr, which are selective but not completely specific for betaproteobacterial ammonia oxidizers followed by a second PCR using the betaproteobacterial primer set CTO189f-GC and CTO654r, as described by Laanbroek and Speksnijder (2008). To detect the bacterial *amoA* gene we used the primer set *amoA*-1F (Nicolaisen and Ramsing, 2002) and *amoA*-2R (Rotthauwe et al., 1997). The GC clamp described by Muyzer and Smalla (1998) was incorporated into the 5′ end of the primers as indicated in **Table 3**.

The final PCR products were separated by DGGE in a 7% polyacrylamide gel with a vertical gradient of 35–60% of formamide and urea denaturants. The running conditions were 100 V at a constant temperature of 60°C for 18 h.

### DGGE-Band Sequencing and Phylogenetic Analysis

The phylogenetic assignment of ammonia-oxidizing prokaryotes sequences was determined by excising the prominent bands from the DGGE gels, eluting in 20 μl of sterile Milli-Q water at room temperature for 2 h. After that, the amplicons were reamplified under the conditions described above and submitted for sequencing at Macrogen Inc., Amsterdam. To identify the closest relatives the obtained sequences were compared with available database using the BLASTn search tool from GenBank^1^. The Bellerophon program (Huber et al., 2004) was used to detect and omit chimeric DNAs.

Phylogenetic relationships of the 16S rRNA gene were inferred with the neighbor-joining algorithm (Saitou and Nei, 1987) with Jukes–Cantor correction (Jukes and Cantor, 1969), using the ARB (version 6.0.1) software package and SSU-Ref-115 database (Ludwig et al., 2004; Pruesse et al., 2007).

For the *amoA* gene, the retrieved nucleotide sequences were aligned to *amoA* gene sequences available on the GenBank^1^ database. The evolutionary history was inferred using neighbor-joining algorithm (Saitou and Nei, 1987) with Jukes–Cantor correction (Jukes and Cantor, 1969). Evolutionary analyses were conducted in MEGA 6 (Tamura et al., 2013). For both genes, the bootstrap consensus tree inferred from 1000 replicates (Felsenstein, 1985) was taken to represent the evolutionary history of the taxa analyzed. The nucleotide sequences generated were deposited into the GenBank database with accession numbers KR028198-KR028271.

### Data Analysis

For the DGGE data, a dendrogram from the fingerprint was calculated using the Dice coefficient of similarity and the unweighted pair-group method with arithmetic averages (UPGMA), within the BioNumerics version 6.5 software package (Applied Maths, Sint-Martens-Latem, Belgium).

Manual selection of environmental parameters was performed after the generation of a Pearson correlation-based heatmap matrix and analysis of the clusters using the free available software packages R^2^. The hierarchical agglomerate clustering was performed using the agglomeration method. Clustering was performed with complete linkage and Euclidean distances as the distance measure.

To determinate multivariate relationships between DGGE banding profiles and environmental parameters we performed a canonical correspondence analysis (CCA), using R software. Moreover, in attempt to correlate the AOA/AOB gene copy numbers ratio with the environmental parameters we performed a principal component analysis (PCA). After standardization of environmental data (by subtracting the mean from each observation and dividing by the corresponding SD), PCA was obtained using the *rda* function in the Vegan library program implemented in R software.

## Results

### Environmental Characterization

**FIGURE 1:**
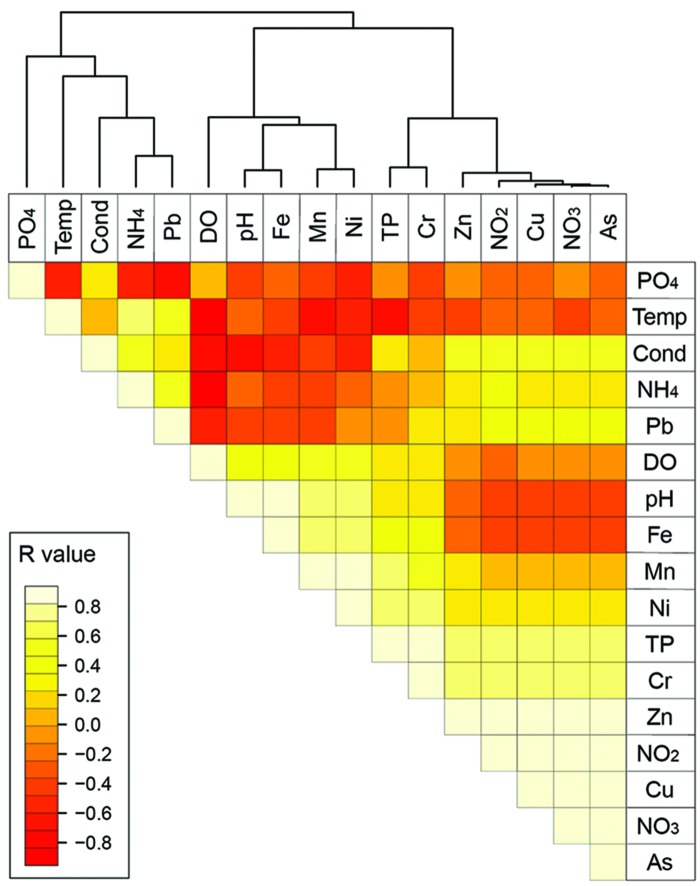
**Heatmap of the environmental parameters correlation**.

A Pearson correlation-based heatmap was generated to arrange the environmental parameters into clusters and correlate them subsequently with the AOA and AOB communities from different sediment samples. The resulting heatmap showed three different clusters: As, NO_3_^-^, Cu, NO_2_^-^, Zn, TP and Cr (cluster 1); Ni, Mn, Fe, pH, and DO (cluster 2); PO_4_^3-^, Pb, NH_4_^+^, temperature, and conductivity (cluster 3; **Figure 1**). From these clusters, NO_3_^-^, TP, Fe, DO, and NH_4_^+^ were chosen for PCA and CCA analyses.

**FIGURE 2:**
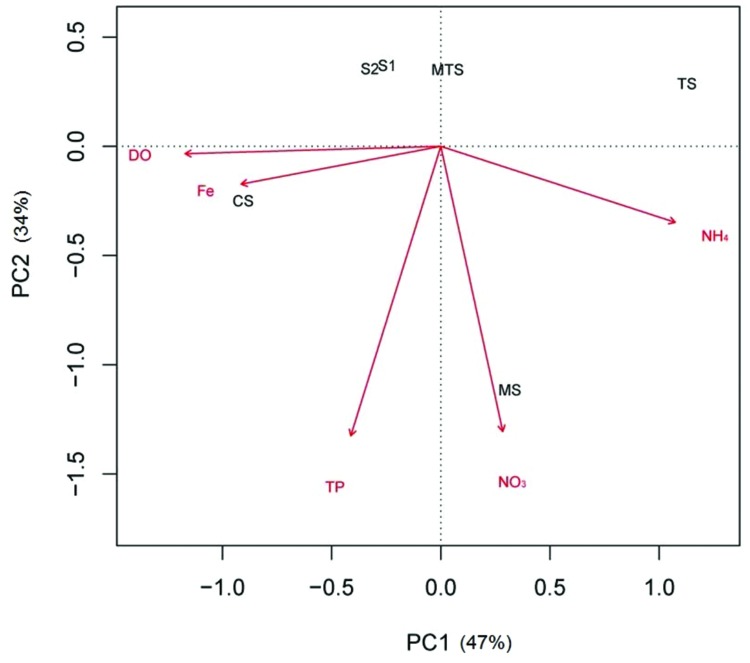
**Principal component analysis ordination biplot of sample locations according to five selected environmental parameters.** CS, Carrapatos sediment; MS, Mina sediment; S1, Site 1 sediment; S2, Site 2 sediment; TS, Tulipa sediment; MTS, Mutuca sediment.

Principal component analysis, which was based on these five environmental parameters, revealed that the first component (PC1) of the PCA biplot, mainly explained the positive correlation of the CS sample with DO and Fe, and a negative correlation with NH_4_^+^, whereas the opposite was observed for the TS sample. The MS sample was determined by the second component (PC2), being mainly positively correlated with NO_3_^-^. The non-impacted sites S1, S2, and MTS were all negatively correlated with NO_3_^-^. In addition, TP and NH_4_^+^ were also negatively correlated, being the first with the MTS sample and the latter with the S1 and S2 samples (**Figure 2**).

### Abundances of AOA and AOB and their Correlation with Environmental Parameters

The absolute abundances of *amoA* gene, i.e., the number of copies of this gene per gram of sediment, were assessed by qPCR. Archaeal *amoA* gene was detected in all samples, whereas for bacteria no *amoA* genes were found in the MTS and S2 samples (**Table 4**). Notably, the S2 sample presented the highest archaeal *amoA* gene copy number/g of sediment (1.4 × 10^5^). Moreover, the copy numbers of bacterial *amoA* genes were higher than those of archaeal *amoA* genes in the CS, MS, and TS samples from mining-impacted sediments (from one to three orders of magnitude). It should be noted that the bacterial *amoA* gene abundance of the MS sample was three orders of magnitude larger than the archaeal.

**Table 4:** Abundance of *amoA* gene of ammonia-oxidizing archaea (AOA) and ammonia-oxidizing bacteria (AOB) and the ratio of AOA over AOB abundances.

Samples	Abundance of AOA *amoA* genes (copies/g of sediment)	Abundance of AOB *amoA* genes (copies/g of sediment)	Ratio of AOA over AOB
MS	506	250000	0.002
CS	24800	110000	0.225
MTS	621	ND	ND
S1	49632	8470	5.85
S2	135281	ND	ND
TS	22	160	0.137

*ND, not detected. MS, Mina stream; CS, Carrapatos stream; MTS, Mutuca stream; S1 and S2, reference sites 1 and 2; TS, Tulipa stream.*

**FIGURE 3:**
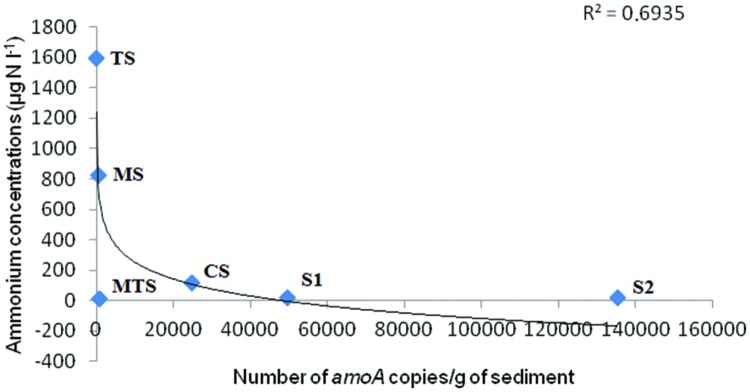
**Correlation between ammonium concentrations in the water and the number of AOA *amoA* copies/g of sediment.** The *r*^2^-values are indicated in the plot.

From the five parameters chosen in the heatmap (**Figure 1**) NH_4_^+^ was only correlated with the archaeal *amoA* gene abundance. As observed in **Figure 3**, the regression line exhibited a negative correlation between the NH_4_^+^ concentrations and AOA *amoA* gene abundance (*r*^2^= 0.6935).

### Analysis of DGGE Banding Patterns

**FIGURE 4:**
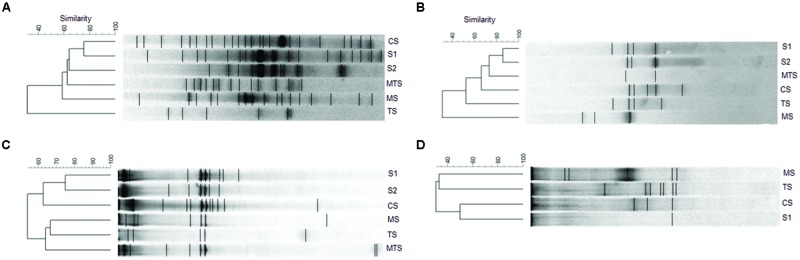
**Unweighted pair-group method with arithmetic averages (UPGMA) cluster analysis of the ammonia-oxidizing archaea (AOA) and ammonia-oxidizing bacteria (AOB) band sequences from the samples. (A)** archaeal 16S rRNA gene; **(B)** archaeal *amoA* gene; **(C)** bacterial 16S rRNA gene; **(D)** bacterial *amoA* gene. CS, Carrapatos sediment; MS, Mina sediment; S1, Site 1 sediment; S2, Site 2 sediment; TS, Tulipa sediment; MTS, Mutuca sediment.

The DGGE banding profiles demonstrated a higher number of bands in the AOA communities than in the AOB communities based on both 16S rRNA and *amoA* genes (**Figure 4**). Moreover, only one bacterial *amoA* gene band was detected in the non-impacted S1 sample, whereas no bands were observed for the AOB community in the non-impacted S2 and MTS samples (**Figure 4D**).

Based on a cut-off of 60% of similarity, the dendrograms based on 16S rRNA DGGE fingerprinting analysis showed rather similar AOA and AOB communities clustering with the mining-impacted sediment sample (CS) grouping with non-impacted sediment samples (**Figures 4A,C**). Moreover, TS and MS were the most dissimilar samples based on archaeal and bacterial 16S rRNA genes, respectively. A similar clustering among the archaeal 16S rRNA and *amoA* genes was also found for the S1, S2, and CS samples (**Figures 4A,B**).

### Phylogenetic Assignment of the AOA Community

**FIGURE 5:**
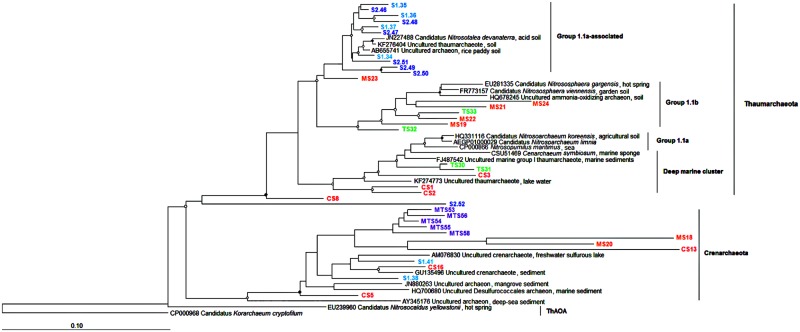
**Neighbor-joining tree of AOA based on the partial 16S rRNA gene of DGGE bands.** Nodes with a bootstrap value greater than 0.90 or 0.50 are indicated by closed and open circles, respectively. CS, Carrapatos sediment; MS, Mina sediment; S1, Site 1 sediment; S2, Site 2 sediment; TS, Tulipa sediment; MTS, Mutuca sediment.

To identify the phylogenetic affiliations of members of the AOA and AOB communities selected bands from the DGGE gels were excised and sequenced. No chimeras were detected among the sequences. For the archaeal 16S rRNA gene, 36 sequences were subjected to phylogenetic analysis (**Figure 5**). It should be noted that all the archaeal sequences from the TS and S2 samples were affiliated with the Thaumarchaeota, whereas the MTS sample, which is oligotrophic and is not influenced by human settlement, was comprised of Crenarchaeota-related sequences. Sequences recovered from the other stream sediments were scattered throughout the Thaumarchaeota and Crenarchaeota phyla. Most sequences from the S1 and S2 samples grouped together in the Group 1.1a-associated clade (**Figure 5**).

From the DGGE fingerprinting profiles of the functional *amoA* gene, 17 sequences were used in the construction of a phylogenetic tree (**Supplementary Figure S1**). Phylogenetic affiliation revealed that the sequences from the CS and MS mining-impacted streams formed a distinct clade (Thaumarchaea group 1.1a) from the non-impacted streams (Group 1.1a-associated). The TS18 sequence was affiliated with *Nitrosopumilus maritimus*, whereas the remaining sequences were affiliated with unclassified archaea. The majority of archaeal *amoA* gene sequences were associated with freshwater sediments sequences present in the GenBank database.

### Phylogenetic Assignment of the AOB Community

**FIGURE 6:**
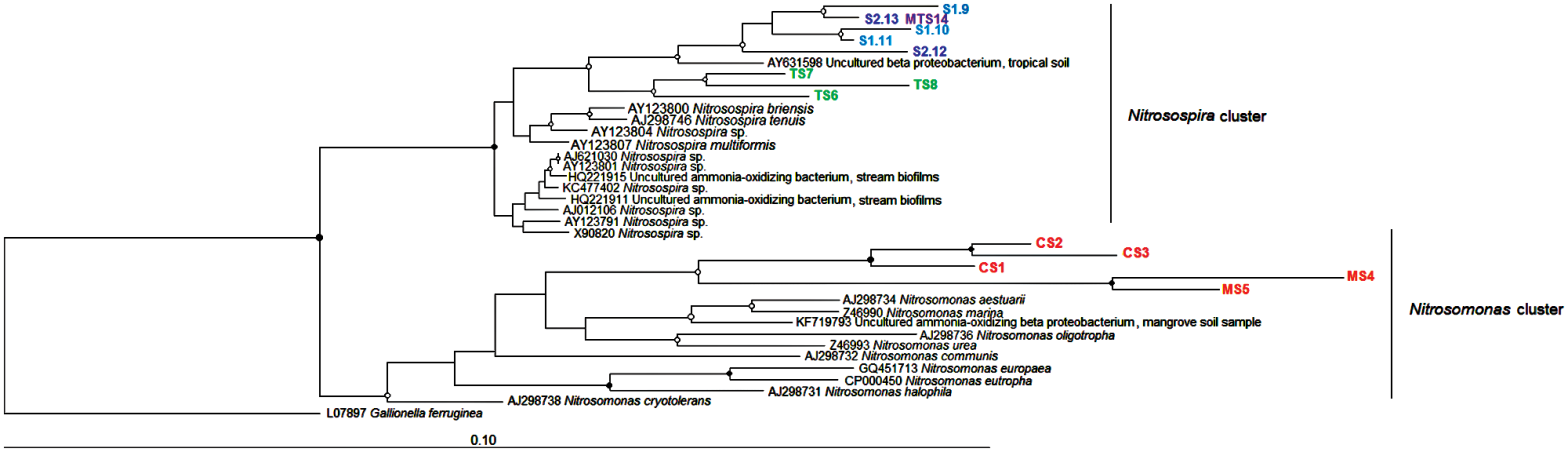
**Neighbor-joining tree of AOB based on the partial 16S rRNA gene of DGGE bands.** Nodes with a bootstrap value greater than 0.90 or 0.50 are indicated by closed and open circles, respectively. CS, Carrapatos sediment; MS, Mina sediment; S1, Site 1 sediment; S2, Site 2 sediment; TS, Tulipa sediment; MTS, Mutuca sediment.

As expected, all the AOB community sequences were affiliated with Betaproteobacteria class. The 14 retrieved betaproteobacterial 16S rRNA-based sequences were allocated phylogenetically in two clades: *Nitrosospira* and *Nitrosomonas* (**Figure 6**). Moreover, the 16S rRNA gene tree revealed an interesting correlation between phylogenetic clustering and trophic state of the environment, i.e., oligo- and ultraoligotrophic (*Nitrosospira*) or meso- and eutrophic (*Nitrosomonas*) sediments.

All the *amoA* gene sequences from the CS, TS, and MS samples did not group into the *Nitrosospira* or *Nitrosomonas* clades (**Supplementary Figure S2**). Furthermore, all *amoA* gene sequences obtained in this study were more closely related to themselves than to reference sequences. Interestingly, sequences from the TS and MS samples, which exhibited high NH_4_^+^ concentrations, were most closely related to sequences recovered from activated sludge and a wastewater treatment plant, respectively. The CS sample sequences were closely related to sequences derived from an estuarine sediment and a freshwater lake.

### Correlation of Ammonia Oxidizers Community Structure with Environmental Parameters

**FIGURE 7:**
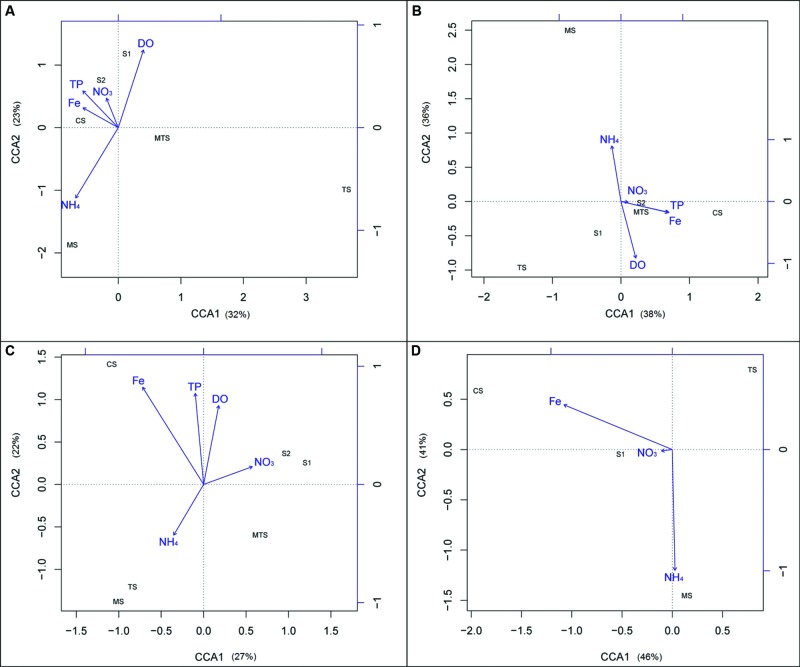
**Canonical correspondence analysis (CCA) ordination diagram of the archaeal and bacterial ammonia-oxidizing communities’ composition data in relation to the five selected environmental variables: **(A)** archaeal 16S rRNA gene; **(B)** bacterial16S rRNA gene; **(C)** archaeal *amoA* gene; **(D)** bacterial *amoA* gene.** CS, Carrapatos sediment; MS, Mina sediment; S1, Site 1 sediment; S2, Site 2 sediment; TS, Tulipa sediment; MTS, Mutuca sediment.

**Figure 7** shows the correlation of the five selected environmental parameters with both AOA and AOB community compositions based on the banding profile of both the 16S rRNA and *amoA* genes. The CCA for the archaeal 16S rRNA gene community showed S2 and CS to be positively correlated with Fe, TP, and NO_3_^-^. Moreover, S1 was positively correlated with DO and negatively correlated with NH_4_^+^, whereas the opposite was observed for the MS community (**Figure 7A**). The bacterial 16S rRNA gene communities from CS, S2, and MTS were positively correlated with Fe, TP, and NO_3_^-^. Again, MS was positively correlated with NH_4_^+^ and negatively with DO, and S1 was positively correlated with DO (**Figure 7B**).

Additionally, using the *amoA* gene banding profile, the AOA communities recovered from the S1 and S2 samples were positively correlated with NO_3_^-^, and MTS was negatively correlated with Fe. Otherwise, the MS and TS communities were positively correlated with NH_4_^+^ and negatively with DO, whereas the CS community was positively correlated with Fe (**Figure 7C**). Regarding to the bacterial *amoA* gene, the TS community was equally explained by both axes CCA1 and CCA2, being negatively correlated with all tested parameters. NH_4_^+^ was the parameter which best explained the segregation of the samples in the plot, and was positively correlated with the MS community. The AOB CS and S1 communities were positively correlated with Fe and NO_3_^-^, respectively (**Figure 7D**).

## Discussion

Many studies have revealed that bacterial and archaeal ammonia-oxidizing communities are shaped by different environmental drivers in many ecosystems (Tourna et al., 2008, 2011; Daebeler et al., 2012; Liu et al., 2014). Overall, our findings demonstrated that distinct environmental characteristics can affect the distribution of AOB communities, clustering them in species-specific niches with *Nitrosomonas* dominating in meso- and eutrophic sediments, and *Nitrosospira* prevailing in oligo- and ultraoligotrophic sediments. Ammonia oxidizers from sediments often experience oxygen depletion due to competition with facultative anaerobes, favoring microaerophilic or anaerobic respiration (Arp et al., 2007). The dominance of *Nitrosomonas*-related sequences from the MS sample could be explained by the inverse correlation between NH_4_^+^ and DO concentrations. Although we were not able to assign the obtained sequences to more narrow defined *Nitrosomonas* and *Nitrosospira* clusters, it is interesting to note for example that the *Nitrosomonas eutropha* genome analysis (IMG, 2006a) revealed genes related to microaerophilic respiration, promoting its growth in environments with oxygen depletion in comparison with *Nitrosospira multiformis*, which lacks these genes in its genome (IMG, 2006b). In addition, it should be highlighted that the *Nitrosospira* and *Nitrosomonas* clades harbor clusters from different environments (Freitag and Prosser, 2003 and references therein). Among these clusters, cluster 7 encompasses the *N*. *eutropha* and *N. europaea* species, which are often recovered from particularly NH_4_^+^-rich environments, such as wastewater treatment plants (Hugenholtz et al., 1998; Herbert, 1999; McCaig et al., 1999). Another interesting characteristic that could have favored the occurrence of *Nitrosomonas* in the CS and MS samples is the capacity of *N*. *eutropha* to cope with exposure to toxic compounds (Arp et al., 2007). Therefore, human settlement activities could favor *Nitrosomonas* over *Nitrosospira*. These findings are in agreement with the study of Dang et al. (2010), who observed that eutrophic sediment with high concentrations of heavy metals favor the predominance of *Nitrosomonas* over *Nitrosospira*, likely due to exogenous input of microbes and nutrients via polluted rivers.

Regarding to AOA phylogeny, it was not possible to obtain a deeper affiliation of the Thaumarchaeota phylum, as all retrieved *amoA* gene sequences were related to unclassified taxa, corroborating with a study in another freshwater system (Vissers et al., 2012). Thus, these sequences could represent novel variants of the *amoA* gene or genes related to unknown ammonia oxidizers. In addition, our findings show that the diversity of *amoA* genes is larger than earlier described, once none *amoA* gene sequence were affiliated with known reference strains. The AOA communities were grouped according to the origin of each sampled sediment, as also reported by Pester et al. (2012) for geographically distinct soils. *Nitrosotalea devanaterra* was formerly described by Lehtovirta-Morley et al. (2011) as an obligate acidophilic ammonia oxidizer (pH from 4 to 5), growing at extremely low ammonia concentration (0.18 nM). In contrast to this report, *N. devanaterra* (**Figure 5** and **Supplementary Figure S1**) was only detected in the non-impacted S1 and S2 sediments characterized by pH 6.5 and NH_4_^+^ concentration of 1,390 nM. Thus, our finding suggests the ability of members of this taxon to occupy NH_4_^+^-rich and circumneutral pH niches, likely due to the presence of novel ecotypes with different physiological characteristics within the *Nitrosotalea* group. *N. devanaterra* was also the most abundant phylotype among the Thaumarchaea encountered in a volcanic grassland soils on Iceland, which had a pH of 6.7 and an NH_4_^+^ concentration of 0.5–1.1 μg per g dry soil (Daebeler et al., 2014). Some members of the soil Thaumarchaeota group 1.1b, such as *Nitrososphaera* found in this study (**Figure 5**), are able to hydrolyze urea leading to an ecological advantage in NH_4_^+^-poor environments (Lu et al., 2012). In the present study the sites are NH_4_^+^-rich, demonstrating the ability of *Nitrososphaera* to deal in environments with a wide range of NH_4_^+^ concentration.

Most of the studies showed that archaeal *amoA* genes are more abundant than bacterial *amoA* genes in many ecosystems (Adair and Schwartz, 2008; Dang et al., 2008; Prosser and Nicol, 2008; Herrmann et al., 2009), with a few reports showing contradictory results in environments such as an activated wastewater treatment bioreactor (Wells et al., 2009), a lake sediment (Jiang et al., 2009), an estuarine sediment (Mosier and Francis, 2008), and now also in mining-impacted freshwater sediments as observed in the present study. In our study, AOB were about one or three orders of magnitude more abundant than AOA in the MS, CS, and TS samples, but not in the S1 sample, suggesting that AOB might be the primary drivers of ammonia oxidation in the sediments impacted by human settlement. Indeed, we did not detect bacterial *amoA* genes in the S2 and MTS samples and rarely in the S1 sample. Auguet et al. (2012) also did not find these genes and reported that it was due to the NH_4_^+^ concentrations in the water, which were below the AOB affinity threshold value, i.e., <1 μM NH_4_^+^ (Bollmann et al., 2002). In our study, S1 and S2 showed a NH_4_^+^ concentration of 1.39 μM, which is slightly higher than this threshold value, whereas the MTS sample had an NH_4_^+^ concentration that was lower than the threshold value (i.e., 0.69 μM). A key finding in our study was that the NH_4_^+^concentration was only inversely proportional to the AOA *amoA* gene abundance and not to the AOB *amoA* gene abundance, likely indicating that lower NH_4_^+^concentrations lead to higher numbers of archaeal *amoA* gene copies, but did not affect the bacterial *amoA* gene copy numbers. Interestingly, Verhamme et al. (2011) reported that high NH_4_^+^ concentrations were proportional to the abundance of bacterial *amoA* gene copies. These findings could suggest that AOA and AOB occupy separate ecological niches, with AOA contributing for the nitrification of ammonia released through mineralization, whereas AOB dominate under high NH_4_^+^ concentrations.

Our data provide insights into the influence of physicochemical parameters of the overlying bulk water on species or ecotypes differentiation of ammonia oxidizer communities in stream sediments. Taken together, our data reveal a different response within the AOB community with *Nitrosomonas* taking advantage over *Nitrosospira* in environments impacted by human settlement. The findings also reveal a high diversity of largely unknown species or ecotypes of the Thaumarchaeota, such as members related to *N. devanaterra* inhabiting ammonia-rich habitats of circumneutral pH values. Moreover, our results demonstrated that the bacterial *amoA* gene was more abundant than the archaeal *amoA* gene in the mining-impacted streams. Regarding that the understanding about the evolutionary history and metabolic repertoire of ammonia oxidizers is still in its beginning, future works would be required to provide additional insights into niche differentiation among these microorganisms.

## Conflict of Interest Statement

The authors declare that the research was conducted in the absence of any commercial or financial relationships that could be construed as a potential conflict of interest.

## Footnotes

1 http://www.ncbi.nlm.nih.gov/

2 http://www.r-project.org

## Supplementary Material

Figure S1 **Neighbor-joining tree of ammonia-oxidizing archaea (AOA) based on the *amoA* gene sequences of denaturing gradient gel electrophoresis (DGGE) bands.** Nodes with a bootstrap value greater than 0.90 or 0.50 are indicated by closed and open circles, respectively. CS, Carrapatos sediment; MS, Mina sediment; S1, Site 1 sediment; S2, Site 2 sediment; TS, Tulipa sediment; MTS, Mutuca sediment.

Figure S2 **Neighbor-joining tree of ammonia-oxidizing bacteria (AOB) based on the *amoA* gene sequences of DGGE bands.** Nodes with a bootstrap value greater than 0.90 or 0.50 are indicated by closed and open circles, respectively. CS, Carrapatos sediment; MS, Mina sediment; TS, Tulipa sediment.
