# Supplementary figures and images for: The effect of human settlement on the abundance and community structure of ammonia oxidizers in tropical stream sediments
Source: Front Microbiol. 2015 Aug 31;6:898. doi: 10.3389/fmicb.2015.00898 (PMC4553384; doi:10.3389/fmicb.2015.00898)

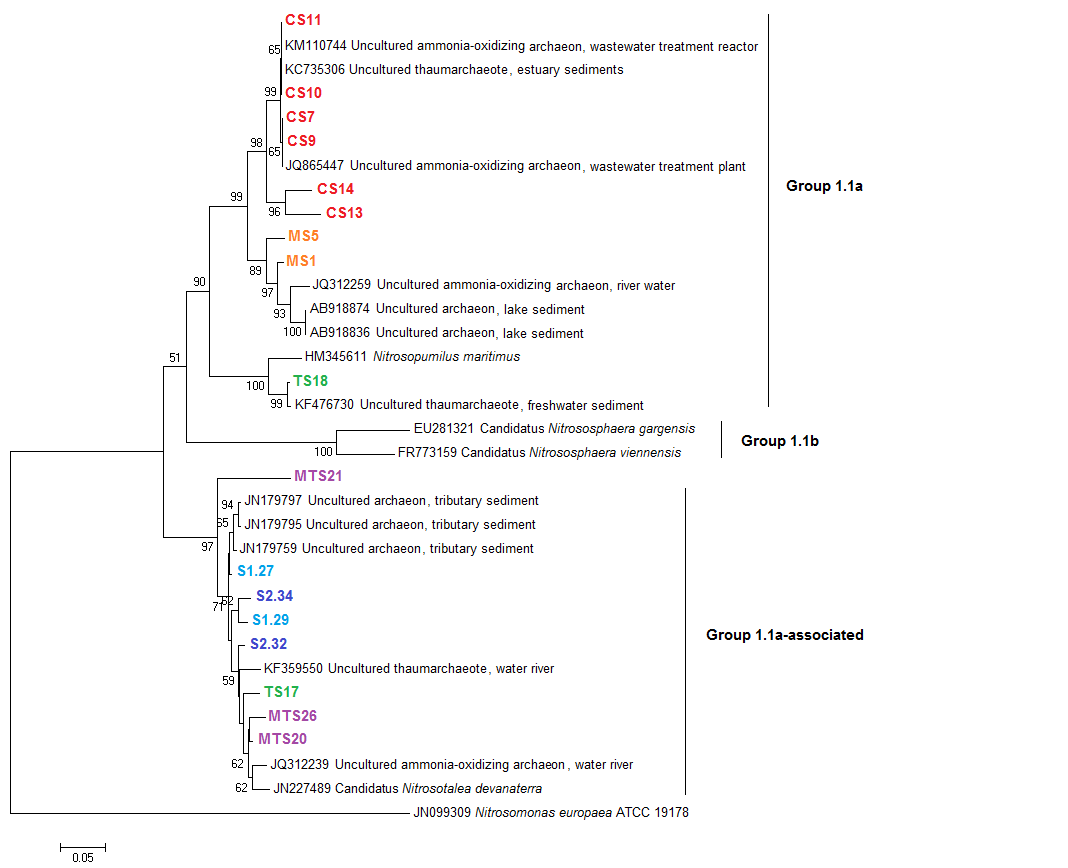

Supplement: Figure S1 — Neighbor-joining tree of ammonia-oxidizing archaea (AOA) based on the amoA gene sequences of denaturing gradient gel electrophoresis (DGGE) bands. Nodes with a bootstrap value greater than 0.90 or 0.50 are indicated by closed and open circles, respectively. CS, Carrapatos sediment; MS, Mina sediment; S1, Site 1 sediment; S2, Site 2 sediment; TS, Tulipa sediment; MTS, Mutuca sediment. [file Image_1.TIF]

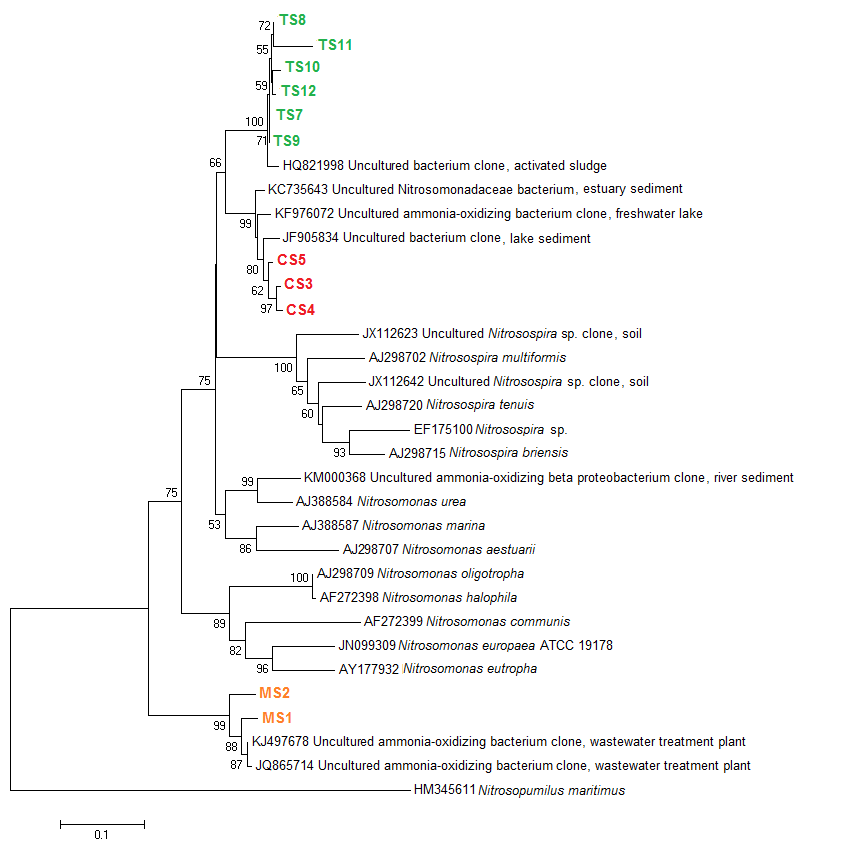

Supplement: Figure S2 — Neighbor-joining tree of ammonia-oxidizing bacteria (AOB) based on the amoA gene sequences of DGGE bands. Nodes with a bootstrap value greater than 0.90 or 0.50 are indicated by closed and open circles, respectively. CS, Carrapatos sediment; MS, Mina sediment; TS, Tulipa sediment. [file Image_2.TIF]
